# Myelin Oligodendrocyte Glycoprotein Antibody-Associated Disease Mimicking Central Nervous System Infection in an Adolescent: A Case Report

**DOI:** 10.7759/cureus.113340

**Published:** 2026-07-25

**Authors:** Panhui Yu, Kang Liu, Fang Chen

**Affiliations:** 1 Department of Pediatric Neurology, Children's Hospital of Hebei Province, Shijiazhuang, CHN

**Keywords:** central nervous system infection, cerebrospinal fluid, diplopia, encephalitis mimic, magnetic resonance imaging, mogad, pediatric neuroimmunology

## Abstract

Myelin oligodendrocyte glycoprotein antibody-associated disease (MOGAD) can resemble central nervous system infection in children because fever, headache, vomiting, cerebrospinal fluid pleocytosis, and multifocal brain lesions may coexist. We report a previously healthy 13-year-old boy who developed headache, fever, vomiting, and binocular horizontal diplopia. Examination showed transient exotropia with apparent adduction limitation but no encephalopathy, seizures, limb weakness, pupillary abnormality, optic neuritis, or spinal cord lesion. Cerebrospinal fluid showed mononuclear-predominant pleocytosis with mildly elevated protein. Brain magnetic resonance imaging showed evolving patchy fluid-attenuated inversion recovery hyperintensities in the right temporal lobe, basal ganglia, periventricular region adjacent to the fourth ventricle, and later the right frontal lobe. Extensive microbiological testing was unrevealing. Serum myelin oligodendrocyte glycoprotein immunoglobulin G (MOG-IgG) was repeatedly positive by live cell-based assay, whereas aquaporin-4 immunoglobulin G (AQP4-IgG) and autoimmune encephalitis antibodies were negative. Clinical improvement occurred during overlapping treatment with intravenous immunoglobulin and corticosteroids. On illness day 97, during prednisone tapering following an individualized off-label decision to use rituximab, the patient remained clinically stable. This case highlights that pediatric MOGAD should be considered when an infection-like central nervous system presentation is accompanied by evolving multifocal magnetic resonance imaging abnormalities and negative pathogen studies. Diplopia can be an important focal clue; however, because the ocular motor examination was incomplete, internuclear ophthalmoplegia could not be confirmed.

## Introduction

Myelin oligodendrocyte glycoprotein antibody-associated disease (MOGAD) is a distinct inflammatory demyelinating disorder of the central nervous system. Diagnosis requires a compatible core demyelinating event, serum myelin oligodendrocyte glycoprotein immunoglobulin G (MOG-IgG) detected with an appropriate cell-based assay, and exclusion of a more plausible alternative diagnosis [1-3]. In children, attacks may include acute disseminated encephalomyelitis, cerebral monofocal or polyfocal deficits, cortical encephalitis, and brainstem or cerebellar syndromes. Fever, headache, vomiting, cerebrospinal fluid pleocytosis, and multifocal brain lesions can make the initial presentation resemble infectious or autoimmune encephalitis [4,5].

This clinical overlap presents a diagnostic challenge. Empirical anti-infective treatment may be appropriate while pathogen studies are pending, but serial reassessment is essential when microbiological testing is unrevealing, and MRI shows a pattern consistent with inflammatory demyelination. In encephalitic MOGAD presentations, MRI may show unilateral, asymmetric, or multifocal cortical, subcortical, and deep gray matter T2/fluid-attenuated inversion recovery abnormalities, which can raise suspicion for inflammatory demyelination in the appropriate clinical context [5,6]. Lesions may also appear, worsen, or persist despite clinical improvement during a single MOGAD attack; this clinicoradiologic mismatch has been termed radiologic lag [6]. MOG-IgG results require similar caution because false-positive results may occur when testing is applied in a low-pretest-probability setting. Current diagnostic criteria favor serum live cell-based assays, whereas fixed-cell or discordant results require careful interpretation in relation to antibody level, clinical phenotype, and pretest probability [1,7].

Diplopia is a useful but nonspecific focal clue in this setting. Brainstem and cerebellar involvement are recognized in MOGAD, and diplopia can occur during symptomatic brainstem or cerebellar attacks [8,9]. However, diplopia alone does not prove MOGAD or establish internuclear ophthalmoplegia. We present a case of pediatric MOGAD that mimicked central nervous system infection and was accompanied by transient ocular motor dysfunction and right-foot thermal dysesthesia. This report highlights the importance of diagnostic integration and cautious interpretation in such cases.

## Case presentation

A previously healthy 13-year-old boy developed intermittent bilateral temporal headache on illness day one. Fever appeared on illness day three. On illness day four, he developed binocular horizontal diplopia, right exotropia, and one episode of vomiting. He was admitted on illness day five. He had no prominent respiratory symptoms, recent vaccination, head trauma, or relevant exposure history. There were no seizures, altered consciousness, behavioral change, speech disturbance, limb weakness, diarrhea, or rash.

On admission, he was alert and afebrile. The pupils were equal and reactive. The right eye was exotropic, and right-eye adduction appeared limited during binocular examination; the left eye initially moved normally. Later during hospitalization, a transient left exotropia with apparent limitation of left-eye adduction was recorded. The available documentation did not establish whether the left-sided finding briefly overlapped with or replaced the right-sided deviation. The ocular misalignment improved within approximately five days. There was no ptosis, anisocoria, vertical ocular motor deficit, meningeal irritation, pyramidal sign, or limb weakness. Bedside testing of pain and temperature sensation was symmetric.

Ophthalmologic assessment documented a 25° exodeviation on synoptophore testing. This finding confirmed objective acute misalignment, but it was not repeated with prism cover testing in different gaze positions. Fusion and stereopsis were absent during the acute misalignment. No abducting nystagmus was observed. Convergence, adducting saccadic velocity, strict monocular ductions, and gaze-dependent incomitance were not documented. As impaired or slowed adduction during horizontal saccades together with abducting nystagmus was not demonstrated, internuclear ophthalmoplegia could not be confirmed. The ocular finding was therefore described as transient ocular motor dysfunction with exotropia and apparent adduction limitation.

Best-corrected visual acuity was 1.0 bilaterally, and comprehensive ophthalmologic evaluation did not support clinically evident optic neuritis or orbital disease.

Lumbar puncture on illness day six showed an opening pressure of 150 mm H2O. Cerebrospinal fluid was clear and colorless, with 195 white cells/µL (reference range, 0-10 cells/µL). Of these, 179 cells/µL (91.8%) were mononuclear, and 16 cells/µL (8.2%) were polymorphonuclear. Protein was mildly elevated at 0.50 g/L (reference range, 0.08-0.43 g/L), whereas glucose was 3.46 mmol/L (reference range, 2.5-4.4 mmol/L) and chloride was 121.8 mmol/L (reference range, 120-132 mmol/L).

Repeat cerebrospinal fluid testing on illness day 20 showed improvement to 39 white cells/µL (reference range, 0-10 cells/µL), including 36 mononuclear cells/µL (92.3%) and three polymorphonuclear cells/µL (7.7%). Protein was 0.31 g/L, glucose was 3.81 mmol/L, and chloride was 118.8 mmol/L.

Serum *Mycoplasma pneumoniae* IgG was positive at 1:160. IgM testing and convalescent paired serology were not available in the clinical record, limiting interpretation of the isolated IgG result. *Mycoplasma pneumoniae* polymerase chain reaction on a respiratory sample and the viral panel were negative. Cerebrospinal fluid metagenomic next-generation sequencing did not detect bacterial, fungal, viral, parasitic, or atypical pathogens. Cerebrospinal fluid stains, cultures, and Epstein-Barr virus and cytomegalovirus polymerase chain reaction were negative. Blood count, C-reactive protein, and procalcitonin were normal. Additional clinical and ancillary evaluations did not support pneumonia or seizure-related encephalopathy.

Serum MOG-IgG was positive by live cell-based assay at 1:32 on illness day eight, after intravenous immunoglobulin had been administered, and was again positive at 1:100 on illness days 22 and 77. Fixed-cell results obtained during serial clinical testing were discordant and were negative on follow-up. As current diagnostic criteria favor serum live cell-based assays, the repeated live-cell positivity in a compatible clinical phenotype was given greater diagnostic weight; the discordant fixed-cell results were interpreted cautiously and did not independently establish or exclude MOGAD. Cerebrospinal fluid fixed-cell testing showed weak MOG-IgG positivity at 1:1 on illness day 8 and was negative on illness day 22. Serum aquaporin-4 immunoglobulin G (AQP4-IgG), glial fibrillary acidic protein-IgG, autoimmune encephalitis antibodies, and systemic autoantibodies were negative. Paired cerebrospinal fluid-serum oligoclonal band testing showed a type 4 matched pattern, with a normal IgG index and IgG synthesis rate. Reiber analysis suggested blood-cerebrospinal fluid barrier dysfunction. These findings were treated as ancillary and nonspecific.

Brain MRI on illness day 8 showed swelling of the medial right temporal lobe and patchy T2/fluid-attenuated inversion recovery hyperintense lesions in the right temporal lobe, right basal ganglia, and periventricular region adjacent to the fourth ventricle. The overall imaging assessment did not support acute ischemia, abscess, tumor, optic nerve involvement, or spinal cord involvement. On illness day 19, right medial temporal swelling had partially improved, while new right frontal T2/fluid-attenuated inversion recovery lesions had appeared. MRI on illness day 30 showed slight local improvement. Illness day 44 imaging showed persistent multifocal right frontal, temporal, and basal-ganglia lesions that were largely unchanged. A separate right frontal sinus cyst was considered incidental and did not explain the multifocal central nervous system abnormalities. Serial axial fluid-attenuated inversion recovery images are shown in Figure 1.

**Figure 1:**
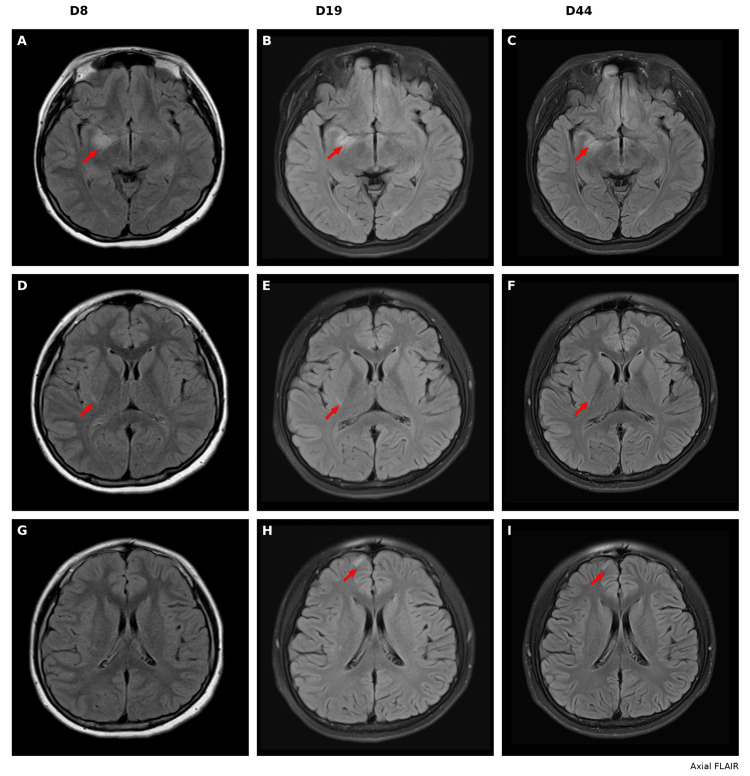
Serial axial fluid-attenuated inversion recovery brain magnetic resonance imaging. Representative axial fluid-attenuated inversion recovery images on illness days eight, 19, and 44 show partial improvement of selected right temporal and basal-ganglia abnormalities with delayed appearance and persistence of a right frontal lesion. Arrows identify representative lesions discussed in the text. The images were obtained during routine clinical care, de-identified, and used with written consent from the patient's legal guardian.

On illness day 16, the patient described reversal of cold and warm sensation in the right foot. The symptom resolved within approximately five days, and repeated bedside examination did not demonstrate a consistent objective sensory deficit. Neurophysiological testing was performed to evaluate the reported thermal dysesthesia and possible peripheral or autonomic involvement. Neurophysiological testing showed preserved lower-limb motor and sensory amplitudes, mildly dispersed waveforms, mildly slowed conduction velocities, and absent lower-limb sympathetic skin responses; other tested modalities were unremarkable. As the symptom was unilateral and brief, the electrophysiological changes were mild and bilateral, and repeat nerve conduction studies were unavailable, this was interpreted as transient sensory symptoms with nonspecific electrophysiological abnormalities rather than confirmed peripheral neuropathy.

The final diagnosis was MOGAD presenting as an infection-like acute central nervous system inflammatory attack with transient ocular motor dysfunction. The diagnosis was based on the acute focal central nervous system syndrome, repeat serum live cell-based assay MOG-IgG positivity, aquaporin-4-IgG negativity, dynamic multifocal brain lesions, including deep gray matter involvement, inflammatory cerebrospinal fluid, and exclusion of more plausible infectious, autoimmune, vascular, neoplastic, and metabolic explanations. Diplopia was considered a focal clinical clue, not an etiologic marker or proof of internuclear ophthalmoplegia.

Acyclovir was administered from illness day five to illness day 18 during the infectious encephalitis evaluation. Azithromycin was given on illness days six to eight because of the isolated *Mycoplasma pneumoniae* serological result. Intravenous immunoglobulin was administered on illness days six and seven at 40 g/day, for a total of 80 g. High-dose intravenous methylprednisolone began on illness day eight at 1,000 mg/day for three days, followed by stepwise dose reduction. Oral prednisone 60 mg/day was started on illness day 20 with supportive prophylaxis.

Rituximab was administered as four 600-mg doses (375 mg/m² per dose, based on a body surface area of 1.60 m²) on illness days 20, 30, 37, and 47 as part of an individualized off-label relapse-prevention strategy discussed with the patient’s guardians. The treating team considered the evolving MRI abnormalities to be concerning for ongoing inflammatory activity. Screening for hepatitis B, hepatitis C, human immunodeficiency virus, and tuberculosis was negative. On illness day 77, B-cell monitoring showed marked depletion, which was interpreted as a pharmacodynamic effect rather than proof of clinical efficacy.

Fever and headache resolved. Diplopia and ocular alignment improved, and by discharge on illness day 22, ocular position and eye movements had largely returned to normal. Cerebrospinal fluid white-cell count decreased from 195 to 39 cells/µL. Prednisone tapering began on illness day 80. At the last available follow-up on illness day 97, the patient remained clinically stable without recurrent diplopia, headache, fever, vomiting, or right-foot thermal dysesthesia. Additional MRI, cerebrospinal fluid testing, and repeat nerve conduction studies had not yet been performed. Longer follow-up was planned to assess relapse risk, MRI evolution, MOG-IgG status, B-cell repopulation, immunoglobulin concentrations, and peripheral nerve outcome. The principal clinical events, investigations, treatments, and outcomes are summarized in Table 1.

**Table 1:** Clinical timeline, investigations, treatment, and outcomes. Illness day 1 was defined as the day of headache onset. Serum MOG-IgG titers shown in the table were measured by a live cell-based assay. The illness day 8 serum sample was obtained after IVIG administration. Abbreviations: CSF, cerebrospinal fluid; IVIG, intravenous immunoglobulin; MOG-IgG, myelin oligodendrocyte glycoprotein immunoglobulin G; MRI, magnetic resonance imaging.

Illness day(s)	Clinical events	Key investigations	Treatment and outcome
Days 1-5	Headache, fever, binocular horizontal diplopia, right exotropia with apparent right-eye adduction limitation, and vomiting; admitted on day 5	Examination showed no encephalopathy, seizures, limb weakness, pupillary abnormality, or meningeal irritation	Acyclovir was started on day 5 and continued through day 18 during evaluation for possible central nervous system infection
Days 6-8	Ocular misalignment persisted and subsequently began to improve	CSF showed 195 white cells/µL with mononuclear predominance and protein of 0.50 g/L; MRI showed right temporal, basal ganglia, and periventricular lesions; serum MOG-IgG was positive at 1:32	IVIG was administered on days 6-7, azithromycin on days 6-8, and intravenous methylprednisolone was started on day 8
Days 16-22	Transient right-foot thermal dysesthesia occurred and resolved; ocular position and eye movements had largely normalized by discharge	Day 19 MRI showed partial improvement of the right temporal lesion and a new right frontal lesion; day 20 CSF white-cell count decreased to 39 cells/µL; day 22 serum MOG-IgG was positive at 1:100	Oral prednisone and the first 600-mg rituximab dose were administered on day 20; the patient was discharged on day 22
Days 30-47	Outpatient follow-up continued	Day 30 MRI showed slight local improvement; day 44 MRI showed persistent multifocal lesions	Three additional 600-mg rituximab doses were administered on days 30, 37, and 47
Day 77	Follow-up assessment	Serum MOG-IgG remained positive at 1:100; B-cell monitoring showed marked depletion	Clinical and laboratory monitoring continued
Days 80-97	No recurrent diplopia, headache, fever, vomiting, or right-foot thermal dysesthesia	No additional MRI, CSF testing, or repeat nerve conduction study was available	Prednisone tapering began on day 80; the patient remained clinically stable at the last follow-up on day 97

## Discussion

This case illustrates a diagnostic challenge highly relevant to pediatric practice. Fever, headache, vomiting, cerebrospinal fluid pleocytosis, and multifocal brain lesions justified the initial evaluation and empirical treatment for possible infection. Repeated pathogen studies were unrevealing, systemic inflammatory markers remained normal, and clinical and ancillary evaluations did not support pneumonia. Moreover, MRI abnormalities evolved despite clinical improvement. In this context, the combination of a focal acute central nervous system syndrome, inflammatory cerebrospinal fluid, evolving multifocal lesions, AQP4-IgG negativity, and repeat serum live cell-based assay MOG-IgG positivity supported MOGAD [1,4-7].

The antibody results were not interpreted in isolation because false-positive MOG-IgG results may occur, particularly when the clinical phenotype or pretest probability is weak [7]. The 2023 international criteria emphasize that diagnosis depends on clinical phenotype, assay method, antibody level, supporting features, and exclusion of more plausible alternatives [1]. This distinction mattered because the first serum sample was obtained after intravenous immunoglobulin and the reports provided quantitative antibody titers rather than internationally standardized clinical categories. Repeat live cell-based assay positivity at later time points increased diagnostic confidence, but persistent MOG-IgG positivity during clinical stability was not treated as evidence of ongoing disease activity by itself [7,10]. Decisions about relapse-prevention therapy were considered separately and individualized.

Internuclear ophthalmoplegia is a clinical syndrome caused by dysfunction of the medial longitudinal fasciculus, which links the abducens nucleus on one side to the contralateral oculomotor nucleus to coordinate horizontal gaze. It is usually supported by impaired or slowed adduction during horizontal saccades and abducting nystagmus, often with supportive brainstem localization [11]. Published MOGAD-associated internuclear ophthalmoplegia cases generally include more specific ocular motor documentation or a corresponding lesion [12,13]. In this patient, no abducting nystagmus was observed, and convergence, saccadic velocity, strict monocular ductions, and gaze-dependent prism measurements were unavailable. The safest description is therefore transient ocular motor dysfunction with exotropia and apparent adduction limitation.

The transient right-foot thermal dysesthesia and mild neurophysiological findings were also not over-labeled. Peripheral nervous system involvement has been described in a small minority of MOGAD patients, mainly as polyradiculopathy [14]. This patient had no weakness, areflexia, radicular pain, reduced nerve amplitudes, or confirmatory follow-up study. The unilateral and brief symptoms did not match the mild bilateral electrophysiological abnormalities. Similarly, isolated *Mycoplasma pneumoniae* IgG did not establish acute infection because IgM, paired serology, respiratory polymerase chain reaction, pneumonia, and pathogen detection in cerebrospinal fluid were absent [15]. The matched oligoclonal band pattern and blood-cerebrospinal fluid barrier dysfunction were nonspecific and did not indicate multiple sclerosis-like intrathecal synthesis [16].

Acute pediatric MOGAD attacks are commonly treated with high-dose corticosteroids, intravenous immunoglobulin, or plasma exchange, depending on severity and response [17]. In this case, improvement occurred after overlapping intravenous immunoglobulin and corticosteroids, so the independent effect of either treatment cannot be determined. Rituximab after a first attack should be viewed cautiously, because maintenance therapy is generally individualized and is more often considered in relapsing disease or severe or incompletely recovering attacks [17]. Observational evidence suggests that rituximab may reduce relapse frequency in some patients but does not eliminate relapse risk [18]. Here, rituximab was used as a case-specific off-label decision in the setting of evolving MRI abnormalities and shared decision-making, not as evidence for routine use after a first pediatric MOGAD attack.

This report has several limitations. First, it describes a single patient with short-term follow-up only, and additional MRI, cerebrospinal fluid testing, and repeat nerve conduction studies were not yet available after illness day 97. Second, the ocular motor examination did not include convergence testing, adducting saccadic velocity, strict monocular ductions, or gaze-dependent prism measurements; therefore, internuclear ophthalmoplegia could not be established. Third, the first serum MOG-IgG sample was obtained after intravenous immunoglobulin, and the laboratory reports did not provide internationally standardized low-positive versus clear-positive categories. Finally, overlapping immunotherapies and rituximab use after a first attack limit any treatment-related inference.

## Conclusions

Pediatric MOGAD should be considered when a child with an infection-like central nervous system presentation has unrevealing microbiological studies and evolving multifocal MRI abnormalities. In this adolescent, the diagnosis was supported by combined clinical, cerebrospinal fluid, MRI, serological, and exclusionary evidence rather than by diplopia, a single antibody result, or treatment response alone. Binocular diplopia was an important focal clue and justified neuro-ophthalmic assessment, but the available examination did not prove internuclear ophthalmoplegia or a specific medial longitudinal fasciculus lesion. Brief thermal dysesthesia and mild electrophysiological abnormalities warranted attention but did not establish confirmed peripheral neuropathy. The practical lesson is the need for disciplined diagnostic integration, in which focal symptoms prompt targeted specialist assessment while isolated infection serology, nonspecific ancillary findings, persistent antibody positivity, and short-term treatment response are interpreted conservatively.
